# Significantly Promoting the Thermal Conductivity and Machinability of Negative Thermal Expansion Alloy via In Situ Precipitation of Copper Networks

**DOI:** 10.1002/advs.202404838

**Published:** 2024-08-28

**Authors:** Minjun Ai, Yuzhu Song, Feixiang Long, Yuanpeng Zhang, Ke An, Dunji Yu, Yan Chen, Yuki Sakai, Masahito Ikeda, Kazuki Takahashi, Masaki Azuma, Naike Shi, Chang Zhou, Jun Chen

**Affiliations:** ^1^ Department of Physical Chemistry Beijing Advanced Innovation Center for Materials Genome Engineering University of Science and Technology Beijing Beijing 100083 China; ^2^ Neutron Scattering Division Oak Ridge National Laboratory Oak Ridge TN 37831 USA; ^3^ Kanagawa Institute of Industrial Science and Technology (KISTEC) 705‐1 Shimoimaizumi Ebina Kanagawa 243‐0435 Japan; ^4^ Laboratory for Materials and Structures Institute of Innovative Research Tokyo Institute of Technology Yokohama 226‐8503 Japan; ^5^ State Key Laboratory for Advanced Metals and Materials University of Science and Technology Beijing Beijing 100083 China; ^6^ Hainan University Haikou Hainan 570228 China

**Keywords:** copper networks, eutectic precipitation, high thermal conductivity, negative thermal expansion

## Abstract

Rapid advancements in electronic devices yield an urgent demand for high‐performance electronic packaging materials with high thermal conductivity, low thermal expansion, and great mechanical properties. However, it is a great challenge for current design philosophies to fulfill all the requirements simultaneously. Here, an effective strategy is proposed for significantly promoting the thermal conductivity and machinability of negative thermal expansion alloy (Zr,Nb)Fe_2_ through eutectic precipitation of copper networks. The eutectic dual‐phase alloy exhibits an isotropic chips‐matched thermal expansion coefficient and a thermal conductivity enhancement exceeding 200% compared with (Zr,Nb)Fe_2_, along with an ultimate compressive strength of 550 MPa. The addition of copper reorganizes the composition of (Zr,Nb)Fe_2_, which smooths the magnetic transition and shifts it toward higher temperature, resulting in linear low thermal expansion in a wide temperature range. The highly fine eutectic copper lamellae construct high thermal conductivity networks within (Zr,Nb)Fe_2_, serving as highways for heat transfer electrons and phonons. The in situ forming of eutectic copper lamellae also facilitates the mechanical properties by enhancing interfacial bonding and bearing additional stress after yielding of (Zr,Nb)Fe_2_. This work provides a novel strategy for promoting thermal conductivity and mechanical properties of negative thermal expansion alloys via eutectic precipitation of copper networks.

## Graphical abstract

A eutectic precipitation strategy is proposed to simultaneously promote the thermal conductivity and machinability of negative thermal expansion alloy Zr_0.7_Nb_0.3_Fe_2_. The copper phase is carefully designed to construct copper networks and contribute additional ductility. The addition of copper also reorganizes composition of Zr_0.7_Nb_0.3_Fe_2_ and results in broadened linear low thermal expansion.

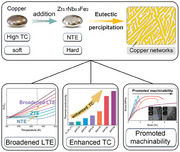

## 1 Introduction

Electronic devices are undergoing design evolution toward increasing integration and miniaturization, leading to a drastic increase in heat generation rate per unit volume. As a result, the lifespan of electronic components decreases significantly with the rapid temperature rise. In addition, the thermal stress caused by a mismatch of coefficient of thermal expansion (CTE) between chips (such as Si, Ge, GaAs, linear CTE of ≈5 ppm K^−1^) and electronic packaging materials will lead to their deformation and even crack.^[^ ^1^, ^2^, ^3^, ^4^ ^]^ Therefore, the design of electronic packaging materials with low CTE and high thermal conductivity is crucial for the development of electronic devices.

Due to inevitable anharmonic lattice vibrations,^[^ ^5^ ^]^ most materials exhibit CTE higher than 10 ppm K^−1^, for example, steels ≈11 ppm K^−1^, aluminum alloys ≈23 ppm K^−1^, copper alloys ≈16 ppm K^−1^, and epoxy ≈69 ppm K^−1^.^[^ ^1^ ^]^ Though great efforts have been made to search for anomalous zero thermal expansion (ZTE) or even negative thermal expansion (NTE) materials,^[^ ^5^, ^6^, ^7^, ^8^, ^9^, ^10^, ^11^, ^12^ ^]^ the range of their thermal conductivities is predominantly below 10 W m^−1^ K^−1^ (e.g., MOF‐5 ∼ 0.3 W m^−1^ K^−1^,^[^ ^13^ ^]^ ZrW_2_O_8_ ∼ 0.7 W m^−1^ K^−1^,^[^ ^14^ ^]^ PbTiO_3_ ∼ 2 W m^−1^ K^−1^,^[^ ^15^ ^]^ La(Fe,Co,Si)_13_ ∼ 6 W m^−1^ K^−1^,^[^ ^16^ ^]^ MnCoGe ∼ 6 W m^−1^ K^−1^,^[^ ^17^ ^]^ Tb(Co_1.9_Fe_0.1_) ∼ 6.3 W m^−1^ K^−1^,^[^ ^18^ ^]^ Invar ∼ 15 W m^−1^ K^−1[^ ^19^ ^]^). A wise strategy for addressing this dilemma is to exploit composites that incorporate both high thermal conductivity materials and low thermal expansion (LTE) materials. Successful explorations have been made for composites of aluminum/graphite,^[^ ^20^ ^]^ copper/graphite,^[^ ^2^, ^21^ ^]^ copper/La(Fe,Co,Si)_13_,^[^ ^3^ ^]^ copper/ZrW_2_O_8_,^[^ ^22^ ^]^ and copper/PbTiO_3_,^[^ ^23^ ^]^ etc. However, the unavoidable porosity and weak bonding force of interfaces between matrixes and reinforcements typically serve as crack initiation under loading, thus constraining the machinability and service life of composites.

Metallic materials generally exhibit superior mechanical properties and the excellent heat transfer by free electrons contributes significantly to their high thermal conductivities.^[^ ^24^, ^25^, ^26^ ^]^ More importantly, in situ multiphase precipitation is enabled in metallic materials and the interfaces between these phases tend to exhibit favorable interfacial bonding. In multiphase alloys, various properties can be modulated,^[^ ^27^, ^28^, ^29^, ^30^, ^31^, ^32^ ^]^ and some challenging trade‐offs can be overcome.^[^ ^33^, ^34^ ^]^ Based on this perspective, the thermal conductivity and machinability of NTE alloys can be promoted if high thermal conductivity alloys are in situ precipitated.

Here, we proposed an innovative eutectic precipitation strategy to significantly promote the thermal conductivity and machinability of NTE alloy Zr_0.7_Nb_0.3_Fe_2_ via in situ precipitation of copper networks. With a moderate introduction of copper at 30 wt.%, the thermal conductivity of the alloy increases by over 200% while the compressive strength reaches 550 MPa. The thermal expansion behavior is regulated to approximate linearity with a chips‐matched CTE in a wide temperature range. The synchrotron X‐ray diffraction and microscopic observations revealed that the alloy consists of highly fine and uniform lamellae of copper and (Zr,Nb)Fe_2_ and shows favorable interfacial bonding, resulting in enhanced mechanical performance. More importantly, lamellae of copper connect as copper networks and significantly promote the thermal conductivity. This work provides an effective strategy for promoting thermal conductivity and machinability of NTE material at a minimum compromise of its NTE, paving the way for NTE alloys’ application as electronic packaging materials.

## 2 Results and Discussion

### 2.1 Eutectic Precipitation Strategy

**Figure 1:**
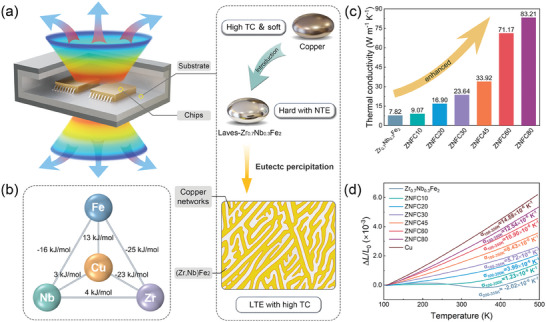
a) Schematic illustration of eutectic precipitation strategy for designing high‐performance electronic packaging materials via in situ forming copper networks in NTE alloy Zr_0.7_Nb_0.3_Fe_2_. (TC represents for thermal conductivity.) b) A demonstration of binary *∆H*_mix_ between Cu, Zr, Nb, and Fe. c) Thermal conductivities of Zr‐Nb‐Fe‐Cu alloys at room temperature. d) Linear thermal expansion curves of Zr‐Nb‐Fe‐Cu alloys compared with pure Cu and Zr_0.7_Nb_0.3_Fe_2_.

Considering the limitations of current design philosophies for developing electronic packaging materials with LTE and high thermal conductivity, an effective design strategy is urgently needed to fulfill demands for higher integration and stronger working capability of electronic devices. A promising approach is to design a dual‐phase alloy that yields in situ forming of high thermal conductivity alloy networks in a NTE matrix (as shown in the schematic diagram in **Figure** **1**a). The networks should serve as a highway for heat transfer electrons as well as phonons and remarkably promote the thermal conductivity.^[^ ^35^, ^36^, ^37^, ^38^, ^39^ ^]^ In fact, eutectic precipitation offers an effective approach for in situ formation of alloy networks.^[^ ^27^, ^40^, ^41^ ^]^ The lamellar eutectic structure enables interconnectivity between these lamellae, thus establishing long‐range highways within the alloy. Following this perspective, we have screened a wide range of both NTE and high thermal conductivity alloys. Among the existing NTE alloys, Zr_0.7_Nb_0.3_Fe_2_ stands out as a promising candidate because of the low fabrication cost and wide‐temperature‐range isotropic NTE.^[^ ^42^, ^43^, ^44^, ^45^ ^]^ Subsequently, to ensure the well‐separated precipitation of the two phases, we analyzed the binary mixing enthalpy (*∆H*_mix_) between typical high thermal conductivity alloys and constituents of Zr_0.7_Nb_0.3_Fe_2_ and selected Cu as the target high thermal conductivity phase. As shown in Figure 1b, it is highly advantageous that the values of *∆H*_mix_ between Fe‐Zr and Fe‐Nb are among the most negative ones while that between Fe‐Cu is the most positive one. It can be expected that Zr_0.7_Nb_0.3_Fe_2_ and copper will precipitate in the solidification process simultaneously. Consequently, a series of Zr‐Nb‐Fe‐Cu alloys denoted as ZNFC10, ZNFC20, ZNFC30, ZNFC45, ZNFC60, and ZNFC80 (the number represents the nominal mass percentage of initial Cu) were synthesized. The proportions of Zr, Nb, and Fe in the alloys follow the same ratio as in Zr_0.7_Nb_0.3_Fe_2_. The thermogravimetric‐differential thermal analysis (TG‐DTA) experiments (Figure S1, Supporting Information) verified the typical eutectic reaction in all the alloys and the synchrotron X‐ray diffraction (SXRD) results confirmed the phase composition of the target (Zr,Nb)Fe_2_ and copper. As is anticipated, the introduction of copper induces the formation of eutectic dual‐phase structure in (Zr,Nb)Fe_2_ and contributes significantly to the thermal and mechanical properties, which will be elaborated below.

### 2.2 Thermal Expansion and Thermal Conductivity Performances

Macroscopic linear thermal expansion and thermal conductivity properties were tested for Zr‐Nb‐Fe‐Cu alloys. It is noteworthy that with the introduction of Cu, thermal expansion behaviors of the alloys are modulated continuously in a roughly linear manner from ZTE to LTE, without disrupting the NTE of Zr_0.7_Nb_0.3_Fe_2_ directly (Figure 1d). The continuity of regulation facilitates the flexible control of the alloy's CTE and enables it to meet diverse CTE requirements in practical applications. By introducing Cu, the thermal conductivities of the alloys are also enhanced by multiples (Figure 1c). ZNFC30, for instance, exhibits a thermal conductivity of 23.6 W m^−1^ K^−1^, marking an enhancement of >200% in comparison with Zr_0.7_Nb_0.3_Fe_2_ (7.82 W m^−1^ K^−1^). In the meanwhile, ZNFC30 maintains an isotropic linear CTE at 5.72 × 10^−6^ K^−1^ over the wide temperature range of 100–350 K (Figure S2b, Supporting Information), close to that of chips. In addition, linear thermal expansion in three orthogonal directions was also examined for ZNFC10 and ZNFC80 (Figure S2, Supporting Information). The thermal expansion curves of each sample show a high degree of concordance in all three directions, demonstrating an isotropic outcome of our eutectic precipitation strategy. When compared with the representative LTE/ZTE/NTE alloys (Table S1, Supporting Information), ZNFC30 exhibits superior incorporation between thermal conductivity and isotropic chip‐matched CTE. In this way, we succeeded in significantly promoting the thermal conductivity of NTE alloy Zr_0.7_Nb_0.3_Fe_2_ and modulating its thermal expansion to wide‐temperature‐range linear behavior with tunable CTE.

### 2.3 Phase and Microstructures

**Figure 2:**
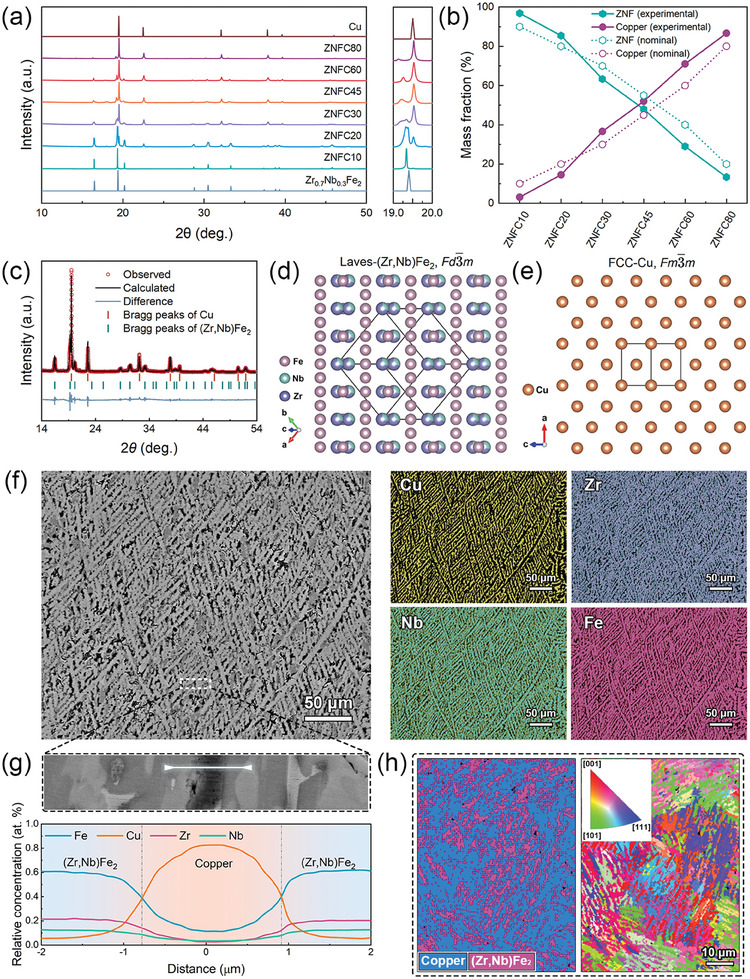
Characterization of phase compositions and phase morphologies of Zr‐Nb‐Fe‐Cu alloys. a) Room‐temperature high‐resolution SXRD patterns of Zr‐Nb‐Fe‐Cu alloys along with the indexed standard cards of FCC‐Cu and Laves‐Zr_0.7_Nb_0.3_Fe_2_. b) Mass fractions of (Zr,Nb)Fe_2_ and Cu phases calculated by Rietveld refinements compared with respective nominal mass fractions. c) A display of structure refinement of SXRD pattern of ZNFC30. d,e) Crystal structures of Laves‐Zr_0.7_Nb_0.3_Fe_2_ and FCC‐Cu, respectively. f) BSE image and corresponding EDS mappings g) Enlarged BSE image focused on a copper lamella (top) and 1D composition profiles across the copper lamella (bottom). h) Phase distribution map (left) and corresponding inverse pole figure (IPF) map (right) showing distribution and grain orientations of Zr_0.7_Nb_0.3_Fe_2_ and Cu phases.

The high‐resolution SXRD was employed to identify the phase composition of all the alloys (**Figure** **2**a). The distinct two sets of peaks correspond to MgCu_2_‐type Laves compound (Zr,Nb)Fe_2_ with space group $Fd\bar{3}m$ and copper with space group $Fm\bar{3}m$, respectively. These alloys can be characterized as composed of the two target phases, which supports the analysis concerning mixing enthalpy above. The SXRD profiles were refined using the Rietveld method to analyze relative phase fraction and the results are shown in Figure 2b and Table S2 (Supporting Information). The phase composition simply exhibits proportional change between (Zr,Nb)Fe_2_ and copper when altering the initial content of Cu. It is reasonable to speculate a simple thermodynamic coexistence relationship between these two phases. The close accordance between actual phase fraction and initial proportion indicates a perfect separation between (Zr,Nb)Fe_2_ and copper phases.

To verify the copper networks in eutectic dual‐phase structure, phase morphologies were observed by scanning electron microscope (SEM). With increasing initial content of Cu in the raw materials, the copper phase underwent a transition from island‐like precipitates to slender dendritic lamellae, then to web‐like branches, and finally to the matrix of (Zr,Nb)Fe_2_ precipitates (Figure S4, Supporting Information). Particularly, for ZNFC30, the alloy exhibits highly fine and uniform lamellar eutectic morphology (Figure 2f). Each lamella in the alloy is ≈100 µm in length with some longer ones extending several hundred micrometers in various directions to connect the lamellae, forming a structure similar to arteries in blood vessels. The width of these lamellae is within only a few micrometers, with lamellae of (Zr,Nb)Fe_2_ being ≈5 µm wide and that of copper ranging from 1 to 3 µm. These lamellae of copper are orderly arranged and interconnect as networks. As suggested above, this structure is expected to facilitate enhanced thermal conductivity performance. Therefore, subsequent investigations in this text will mainly focus on ZNFC30. Furthermore, the general width of the copper lamellae can be visually determined from its representative magnified BSE image (Figure 2g), while the width of the interface between two phases can be observed from 1D compositional profiles across copper lamella, which is <1 micron. Phase composition can be identified as Zr_21_Nb_11_Fe_62_Cu_6_ (at. %) for (Zr,Nb)Fe_2_ and Cu_92_Fe_6_Nb_2_ (at. %) for copper phase according to energy dispersive spectrometer (EDS) results. Additionally, the isotropic grain feature of ZNFC30 was evidenced by electron back scatter diffraction (EBSD) experiments (Figure 2h) from which no preferential orientation was observed.

**Figure 3:**
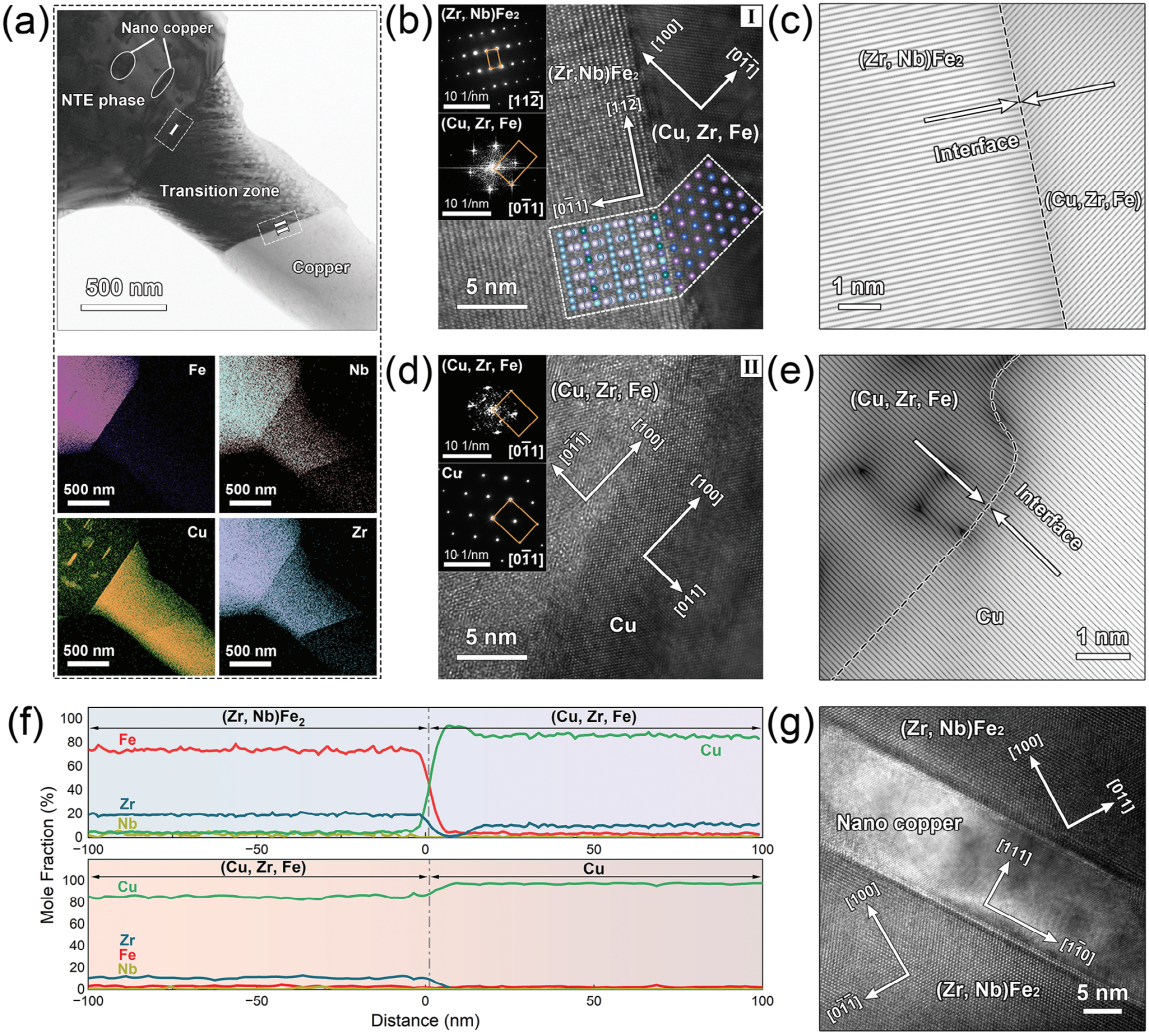
Detailed microstructure of ZNFC30 observed by TEM. a) DF‐TEM image focused on the interface between (Zr,Nb)Fe_2_ and copper phases (top) and corresponding EDS mappings (bottom). b) HRTEM image of the interface between (Zr,Nb)Fe_2_ and transition zone. Insets in the upper left are respective SAED patterns and the inset in the lower right shows atomic interfacial relationship on the interface. c) IFFT image of (Zr,Nb)Fe_2_ along [11$\bar{2}$] direction and transition zone along [100] direction. d) HRTEM image of the interface between transition zone and copper. Insets in the upper left are respective SAED patterns. e) IFFT image of the transition zone and copper along [100] direction. f) 1D composition profiles across the interface between (Zr,Nb)Fe_2_ and transition zone (top) and the interface between the transition zone and copper (bottom). g) HRTEM image of nano copper.

The detailed microstructure of ZNFC30 was further revealed by in‐depth transmission electron microscope (TEM) analysis. A representative (Zr,Nb)Fe_2_‐transition zone‐copper structure can be detected from the Dark Field (DF)‐TEM image and corresponding EDS mappings near the interface between these two phases (**Figure** **3**a). On the basis of TEM results, elements of Fe, Zr, and Nb are mainly partitioned in the (Zr,Nb)Fe_2_ region with composition identified as Zr_19.8_Nb_10.5_Fe_66.2_Cu_3.5_ (at. %). In the copper phase, the content of Zr, Nb, and Fe is minimal while Cu is the predominant constituent. The transition zone, several hundred nanometers in width, mainly consists of Cu and Zr. Chemical compositions are identified as Cu_97.7_Fe_2.3_ (at. %) and Cu_83.0_Zr_10.1_Fe_4.6_Nb_2.3_ (at. %) for the copper phase and transition zone, respectively. It can be inferred based on the traditional eutectic solidification theory^[^ ^46^, ^47^, ^48^ ^]^ that preferential precipitation of (Zr,Nb)Fe_2_ lamellae occurs first during the solidification process due to the more negative *∆H*_mix_ between Zr, Nb, and Fe. Subsequently, in the vicinity of (Zr,Nb)Fe_2_ lamellae, where concentrations of Zr, Nb, and Fe decrease, the transition zone precipitates due to negative *∆H*_mix_ between Cu and Zr, followed by precipitation of copper phase. As the concentration of Cu decreases, the precipitation of (Zr,Nb)Fe_2_ lamellae continues. This cyclic process results in eutectic phases finally. It is worth noting that before the precipitation of the copper phase, remaining Zr and Fe are largely concentrated in the transition zone, which purifies the copper phase and thus promises its thermal conductivity by reducing the scattering of heat transfer electrons.

Furthermore, as interfaces are essential for the thermal and mechanical properties of multiphase alloys, comprehensive investigations were carried out to gain insight into interfacial bonding of (Zr,Nb)Fe_2_‐transition zone‐copper structure. High‐resolution transmission electron microscopy (HRTEM) images of the interface between (Zr,Nb)Fe_2_ and transition zone, as well as the interface between transition zone and copper are presented in Figure 3b,d, respectively. SAED patterns (insets in the upper left of Figure 3b,d) confirm the MgCu_2_‐type structure of (Zr,Nb)Fe_2_ and FCC structure of both transition zone and copper phase, indicating that the transition zone is a copper solid solution, denoted as (Cu,Zr,Fe). Due to differences in crystal structure symmetries between (Zr,Nb)Fe_2_ and transition zone, they adopt the orientation relationship that (111) planes of both sides are parallel to their interface (as illustrated in the schematic atomic diagram in the lower right of Figure 3b), aiming to minimize the interfacial energy. Lattice misfit (*δ*) can be calculated by *δ* $=\;2(\frac{\sqrt{2}}{2}a_{(\mathrm{Zr},\mathrm{Nb})Fe_{2}}\;-\;\frac{\sqrt{6}}{2}a_{\left(\mathrm{Cu},\mathrm{Zr},\mathrm{Fe}\right)})/(\frac{\sqrt{2}}{2}a_{(\mathrm{Zr},\mathrm{Nb})Fe_{2}}+\frac{\sqrt{6}}{2}a_{\left(\mathrm{Cu},\mathrm{Zr},\mathrm{Fe}\right)})$ = 11%, where lattice parameters $a_{(\mathrm{Zr},\mathrm{Nb})Fe_{2}}$ and *a*_(Cu,Zr, Fe)_ were determined from HRTEM images as 7.128 and 3.679 Å, respectively. An atypical coincidence site lattice (CSL) with a multiplicity of ≈10 is formed along the interface (as shown in inverse fast Fourier transform (IFFT) of ${\left(2\bar{2}0\right)}_{(\mathrm{Zr},\mathrm{Nb})Fe_{2}}$ and (200)_(Cu,Zr, Fe)_ in Figure 3c). The interface between the transition zone and copper phase is not so distinct (Figure 3d–f). The two copper solid solutions take the same orientation and lattice misfit *δ* can be calculated as δ  =  2(*a*_(Cu,Zr, Fe)_  − *a*_copper phase_)/(*a*_(Cu,Zr, Fe)_  +  *a*_copper phase_) = 1.3%, where *a*_copper phase_ was determined to be 3.630 Å. A coherent interface with few misfit dislocations extending into the transition zone can be observed from the IFFT of (100) plane across the solid solutions in Figure 3e. In general, interfaces in (Zr,Nb)Fe_2_‐transition zone‐copper structure are well bonded and help impede dislocation nucleation and shear sliding,^[^ ^49^, ^50^ ^]^ contributing enhancements to the mechanical properties of the alloy. Additionally, nano‐copper particles identified as Cu_50.6_Fe_38.0_Zr_10.2_Nb_1.2_ (at. %) FCC solid solution were spotted within (Zr,Nb)Fe_2_ phases (Figure 3a,g). Nano‐copper and (Zr,Nb)Fe_2_ matrix share the same orientation relationship exhibited by (Zr,Nb)Fe_2_ and transition zone. It can be expected that these soft nano‐copper particles would improve both the strength and ductility of the (Zr,Nb)Fe_2_ matrix.^[^ ^51^, ^52^ ^]^

### 2.4 Magnetic Structure and LTE Mechanism

**Figure 4:**
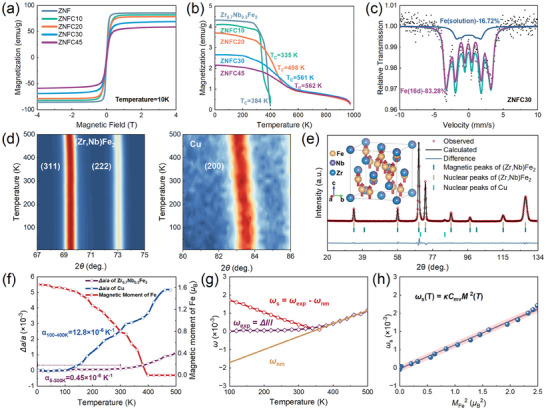
Macroscopic magnetic properties of Zr‐Nb‐Fe‐Cu alloys and magnetic ZTE of ZNFC10 determined by temperature‐dependent NPD a) Magnetization curves as a function of magnetic field at 10 K. b) Field cooling temperature‐dependent magnetization curves measured under an external magnetic field of 0.01 T. c) Mössbauer spectrum of ZNFC30 acquired at 6.2 K. d) Change of peak intensities of (Zr,Nb)Fe_2_ (left) and copper (right) with increasing temperature. e) A display of magnetic structure refinement of NPD pattern at 5 K. Inset in upper left illustrates crystal and magnetic structure of (Zr,Nb)Fe_2_. The red arrows represent the magnetic moments of Fe atoms. f) Temperature‐dependent magnetic moment of (Zr,Nb)Fe_2_ and relative change of lattice parameters of (Zr,Nb)Fe_2_ and Cu calculated from refinement results. g) Temperature dependence of spontaneous magnetostriction coefficient (*ω*_s_) calculated by Debye–Grüneisen model. h) Proportional relationship between *ω*_s_ and *M*_Fe_^2^. The equation of *ω*_s_ is presented in the upper left, where *κ* is compressibility and *C*_mv_ is the magnetovolume coupling parameter.^[^ ^56^, ^57^ ^]^

With increasing copper content, the NTE of Zr_0.7_Nb_0.3_Fe_2_ is modulated continuously to LTE. Therefore, it is necessary to investigate the influence of copper addition on the magnetic structure of the alloys. First, macroscopic magnetic behaviors of Zr_0.7_Nb_0.3_Fe_2_, ZNFC10, ZNFC20, ZNFC30, and ZNFC45 alloys were tested. The dependence of magnetization on applied magnetic field at temperature of 10 K (shown in **Figure** **4**a) confirmed the ferromagnetism of all the alloys. It is known that Cu atoms with fully occupied 3d orbitals are non‐magnetic, therefore, the addition of copper results in a gradual decrease of saturation magnetization of the alloys compared with single Zr_0.7_Nb_0.3_Fe_2_. To determine the intrinsic magnetic transition behavior, field cooling temperature dependence of magnetization was measured at a low external magnetic field of 100 Oe (Figure 4b). A typical transition from ferromagnetism to paramagnetism was observed for each alloy. Furthermore, it can be found that except for ZNFC10, Curie temperature (*T*_c_) of other alloys is growing larger with increasing copper content (Figure 4b). Based on TEM results, the addition of Cu mainly exerts two impacts on the microstructure of Zr_0.7_Nb_0.3_Fe_2_. First, a small amount of Cu atoms occupy the 16d site of Zr_0.7_Nb_0.3_Fe_2_ as substitutional atoms and thus weaken the magnetic exchange interaction between Fe‐Fe atoms. Second, the eutectic copper phase absorbs parts of Zr and Nb atoms from Zr_0.7_Nb_0.3_Fe_2_, resulting in a deviation of the ratio of Zr/Nb from 7:3. When the copper content is relatively low, ZNFC10 for example, Cu mainly exist as substitutional atoms and cause decrease of *T*_c_. When Cu reaches its substitution limit in (Zr,Nb)Fe_2_, the solution of Zr and Nb in copper will be more pronounced, which modifies the magnetic interaction in (Zr,Nb)Fe_2_. As a result, the magnetic transition of (Zr,Nb)Fe_2_ becomes smoother and shifts to higher temperatures, leading to LTE in a wide temperature range. The smoothing of magnetic transition is reflected in the Zr‐Nb‐Fe‐Cu alloys’ decrease of changing rate of magnetization with temperature compared with Zr_0.7_Nb_0.3_Fe_2_ (Figure 4b). This smoothing results in the linear thermal expansion curves since it is believed that the decreasing rate of magnetization determines the contribution of magnetic NTE.^[^ ^7^ ^]^ Additionally, the solution of Zr and Nb in copper leads to an excess of Fe in (Zr,Nb)Fe_2_. As a result, excessive Fe atoms are forced to precipitate as BCC solid solution,^[^ ^53^, ^54^ ^]^ which is supported by the Mössbauer spectrum acquired on ZNFC30 (Figure 4c). The two sub‐spectrums with magnetic hyperfine splitting characteristics correspond to Fe in the 16d site and excessive BCC‐Fe, respectively. The existence of BCC‐Fe accounts for high‐temperature‐stable magnetization enduring until 973 K when the initial mass fraction of Cu exceeds 20% (Figure 4b).

In addition, We have calculated the electron density of states (DOS) of Zr_0.75_Nb_0.25_Fe_2_ and Zr_0.75_Nb_0.25_(Fe,Cu)_2_ (Figure S8, Supporting Information) to investigate the impact of copper addition on the magnetic structure of (Zr,Nb)Fe_2_ theoretically. Although the DOS of the spin up and spin down electrons of Cu are asymmetrical, the integrated spin up and spin down electron numbers of Cu in Zr_0.75_Nb_0.25_(Fe,Cu)_2_ are 6.18 and 6.21 a.u., respectively. The almost equal value elucidates the non‐magnetic behavior of Cu. Furthermore, the DOS of Fe in Zr_0.75_Nb_0.25_(Fe,Cu)_2_ is lower than that in Zr_0.75_Nb_0.25_Fe_2_, which suggests the weakened magnetism of Zr_0.75_Nb_0.25_Fe_2_ unit cell. As for the shifting of magnetic transition toward higher temperature, the previous work^[^ ^45^ ^]^ has interpreted through theoretical calculations that the higher Zr/Nb ratio will lead to longer Fe‐Fe distance. Accordingly, the exchange interaction between the magnetic atoms of Fe will be enhanced and the Curie temperature will increase.

Furthermore, temperature‐dependent neutron powder diffraction (NPD) was carried out on ZNFC10 to further investigate the magnetic structure. Distinct ZTE and positive thermal expansion (PTE) behaviors can be distinguished respectively from peak shifts of ${\left(311\right)}_{\left(\mathrm{Zr},\mathrm{Nb}\right)Fe_{2}}$ and (200)_Cu_ toward low diffraction angles (Figure 4d). The diffraction patterns were well refined with Cu ($Fm\bar{3}m$) and magnetic (Zr,Nb)Fe_2_ ($Fd\bar{3}m$) phases (Figure 4e). The temperature‐dependent lattice parameters of (Zr,Nb)Fe_2_ and Cu, as well as magnetic moments of Fe atoms are shown in Figure 4f. According to refinement results, the copper phase exhibits rough linear PTE with CTE of 12.8 × 10^−6^ K^−1^ at the temperature range of 100–400 K, while (Zr,Nb)Fe_2_ exhibits ZTE with CTE of 0.45 × 10^−6^ K^−1^ from 300 K down to zero temperature. The occupation of Cu in the 16d site is calculated as 6.86% (Table S4, Supporting Information). As is analyzed above, magnetic interactions between Fe‐Fe atoms are weakened by the substitution of Cu, resulting in diminished magnetic NTE. To make a quantitative description of magnetic contribution to thermal expansion, spontaneous magnetostriction (*ω*_s_) of (Zr,Nb)Fe_2_ phase was calculated by subtracting non‐magnetic parts (*ω*_nm_, mainly contributed by vibrations of phonons) from experimental lattice thermal expansion (*ω*_exp_, *∆a/a* from NPD results), where *ω*_nm_ is calculated by extrapolating paramagnetic parts of *ω*_exp_ based on Debye–Grüneisen model (as shown in Figure 4g).^[^ ^55^ ^]^ Similar to Zr_0.7_Nb_0.3_Fe_2_, the proportional relationship between *ω*_s_ and square of local magnetic moments (*M*(*T*)^2^) also applies for (Zr,Nb)Fe_2_ in ZNFC10 (Figure 4h), which indicates the well‐remained magnetic NTE of (Zr,Nb)Fe_2_.

### 2.5 Mechanical Properties and Co‐Deformation Mechanism

**Figure 5:**
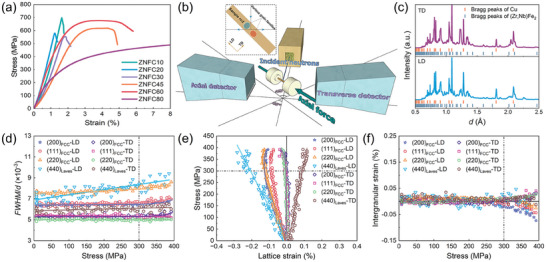
Macroscopic compressive mechanical properties of Zr‐Nb‐Fe‐Cu alloys and results of real‐time in situ neutron diffraction experiments under axial compressive stress of ZNFC30 calculated by SPF method. a) Compressive engineering stress‐strain curves of Zr‐Nb‐Fe‐Cu alloys. b) Schematic diagram of real‐time in situ neutron diffraction experiments under axial compressive stress. c) Neutron diffraction patterns of ZNFC30 along TD and LD under zero stress in the real‐time in situ neutron diffraction experiments. d) *FWHM/d* of (Zr,Nb)Fe_2_ and copper under applied stress. e) Microscopic lattice strains of (Zr,Nb)Fe_2_ and copper under applied stress. f) Intergranular strain between (Zr,Nb)Fe_2_ and copper.

Mechanical performance is the ultimate criterion for evaluating a potential candidate for packaging material. Macroscopic compressive tests were conducted on all series of Zr‐Nb‐Fe‐Cu alloys, given that packaging materials are typically involved with compressive loading. As expected, the alloys investigated exhibit favorable mechanical properties in ultimate compressive strength and fracture strain (**Figure** **5**a). From the evolution of engineering stress–strain curves, it can be found that besides the significant promotion of thermal conductivity, the introduction of ductile copper also substantially enhances the plasticity of Zr‐Nb‐Fe‐Cu alloys. For comparison, the intermetallic compound of Zr_0.7_Nb_0.3_Fe_2_ is intrinsically brittle due to mixed ionic, metallic, and covalent bonding, as well as lacking enough slip systems.^[^ ^58^, ^59^ ^]^ Fracture always occurred before the initiation of slip systems starts when it is subjected to compressive loading. With increase in copper content, for ZNFC10, ZNFC20, and ZNFC30, fracture strain is effectively improved with a slight decrease of ultimate compressive strength from 700 MPa, but they are still brittle alloys without yielding before fracture. For ZNFC45 and ZNFC60, these alloys transform into ductile behavior, and an increase in copper content leads to an ultimate compressive strength to 685 MPa due to the work hardening. In the meanwhile, a steady microplastic deformation stage emerges after the elastic limit and extends with larger copper fraction, which optimizes fracture strain and plasticity of the alloys. Finally, a ductile stress‐strain curve was obtained for ZNFC80 (Figure S10, Supporting Information), which is characterized by a continuous strain‐hardening effect until its final fracture at the strain of 41.5%.

The evolution of mechanical properties can be largely correlated with the variation of phase microstructures.^[^ ^27^, ^40^, ^41^ ^]^ As is revealed by SEM observations, the Zr‐Nb‐Fe‐Cu alloys involve brittle (Zr,Nb)Fe_2_ phase and ductile copper phase. For ZNFC10 and ZNFC20, the minority phase of copper is embedded on (Zr,Nb)Fe_2_ matrix, additional slip systems brought by copper can contribute to the deformation of the alloys but it is relatively limited. For ZNFC30, eutectic lamellae of (Zr,Nb)Fe_2_ and copper are formed and significantly influence the co‐deformation mechanism. Especially for ZNFC60, the increase of soft copper phase has attained sufficient fraction to bear the majority phase‐specific stress and turn the brittle alloy into a ductile one. When copper content is large enough, (Zr,Nb)Fe_2_ serves as dispersive strengthening precipitates in the copper matrix and endows the alloys with satisfactory strength and remarkable strain‐hardening capability.

To gain insight into the co‐deformation mechanism between (Zr,Nb)Fe_2_ and copper lamellae in the eutectic alloys, real‐time in situ neutron diffraction experiments under axial compressive stress were carried out on ZNFC30 (Figure 5b,c). Stress‐dependent phase‐specific lattice strains of various grain families oriented toward loading direction (LD) and transverse direction (TD), as well as the full width at half maximum (*FWHM*) of corresponding peaks were extracted using single peak fitting (SPF) method.^[^ ^60^, ^61^ ^]^ Furthermore, grain‐specific intergranular strain *ε_hkl‐_*_inter_ was calculated by *ε_hkl‐_*_inter_ = *ε_hkl_* – *σ*/*E_hkl_* for positive *ε_hkl_* and *ε_hkl‐_*_inter_ = *σ*/*E_hkl_* – *ε_hkl_* for negative *ε_hkl_*,^[^ ^60^ ^]^ where *ε_hkl_* is *hkl‐*oriented lattice strain, *σ* is the applied stress, and *E_hkl_* is diffraction elastic constant, the slope of initial linear stress‐lattice strain curve, which reflects crystalline elastic anisotropy. Here *ε_hkl‐_*_inter_ can be understood as a deviation of lattice strain from initial linear change. Based on the variations of lattice strains and *FWHM*s over the applied stress, the deformation process can be divided into two stages. When the stress is below 300 MPa (Stage I, elastic strain‐free deformation), grain families of both phases exhibit linear dependence of lattice strains on applied stress, indicating an absence of intergranular strains. In the meanwhile, the little dependence of *FWHM/d* on applied stress (Figure 5d) suggests the elastic behavior of these grain families, because *FWHM*/*d* is affected by development of local strain mostly due to dislocation densities in the in situ loading process.^[^ ^62^ ^]^ However, there are two exceptions that Both (220)_FCC_‐LD and (440)_Laves_‐LD grains indicate measurable increase of *FWHM*/*d* v.s. applied stress, which accounts for larger increments of their lattice strain in Figure 5e. Consequently, the strain of copper in the transverse direction is constrained by hard Laves lamellae that bear more strain in LD and TD. When the applied stress exceeds 300 MPa (Stage II, yielding of FCC phase), *FWHM*/*d* of FCC grains in LD start to increase, indicating the yielding of FCC phase. In this stage, as the hard Laves phase keeps rapid strain increments and continues to bear more strain, FCC copper begins to show negative intergranular strain after yielding, which means stress of FCC copper are partially transferred to Laves phase. Therefore, for ZNFC30 in the loading process, the Laves phase is stress‐bearing and enhances the mechanical strength of the alloy, while FCC copper facilitates the plastic deformation.

## 3 Conclusion

In summary, we proposed an effective strategy to promote the thermal conductivity and mechanical performances of NTE alloy (Zr,Nb)Fe_2_ via in situ precipitation of copper networks. With the addition of copper, partial Cu substitutes Fe and a few Zr and Nb dissolved in Cu. Due to the recombination of (Zr,Nb)Fe_2_ components, magnetic interactions are modulated, resulting in a smoother magnetic transition and a shift toward higher temperature. As a result, the LTE of Zr‐Nb‐Fe‐Cu alloy can be modulated continuously in a nearly linear manner over a wide temperature range. In addition, incorporation between lamellae of hard (Zr,Nb)Fe_2_ and ductile copper, as well as favorable interfacial bonding between these lamellae significantly facilitate the mechanical performances of Zr‐Nb‐Fe‐Cu alloys. More importantly, web‐like arranged copper lamellae significantly enhance the thermal conductivity of the alloys. Additionally, if other systems share similar thermodynamic relationships with a high thermal conductivity alloy to (Zr,Nb)Fe_2_, there may also be a tendency to form eutectic alloys. It only needs to focus on the structure of eutectic phases and to search for systems capable of forming interconnected high thermal conductivity alloy networks. In this way, we developed an effective approach to significantly promote the thermal conductivity and mechanical properties of NTE alloys, shedding light on their practical application as electronic packaging materials.

## 4 Experimental Section

### Alloys Preparation

The ingots of Zr‐Nb‐Fe‐Cu alloys were prepared by arc melting under an argon atmosphere from commercial high‐purity metals (Cu 99.99%, Fe 99.99%, Zr 99.95%, Nb 99.95%). To ensure homogeneity, each sample was melted four times with turnover and subsequently annealed in a vacuum‐sealed quartz tube at 1073 K for three days. Various sizes of samples were fabricated from these annealed alloys for subsequent tests.

### Determination of Phase Composition and Microstructure

The high‐resolution synchrotron X‐ray diffraction (SXRD) patterns were collected at the BL02B2 beamline of SPring‐8 to analyze the phase composition and crystal structure. Thermogravimetric‐differential thermal analysis (TG‐DTA) combined thermoanalytical technique was employed to examine the melting/solidification process. Observations of distribution, morphologies, as well as orientation relationship of the phases were conducted on ZEISS GeminiSEM 500 scanning electron microscope (SEM) equipped with energy dispersive spectrometer (EDS) and electron back scatter diffraction (EBSD) probes. Further microscopic structure was identified by FEI Tecnai F30 transmission electron microscope (TEM).

### Measurements of Physical Performances

The macroscopic linear thermal expansion curves were measured by NETZSCH DIL 402 Expedis thermal dilatometer, and thermal conductivities were measured on a CTM‐60 thermal conductivity meter with each sample measured three times for error reduction. Additionally, to assess the mechanical properties of the alloys, compression tests were conducted on MTS E44.304 electromechanical test systems. Microscopic co‐deformation mechanism was revealed by real‐time in situ neutron diffraction experiments under axial compression loading, which were conducted at VULCAN BL‐7 engineering materials diffractometer at the Spallation Neutron Source, Oak Ridge National Laboratory (ORNL).

### Characterization of Magnetic Properties and Magnetic Structure

The macroscopic magnetic tests were conducted on a physical property measurement system (PPMS) by Quantum Design. Temperature‐dependent neutron powder diffraction (NPD) profiles were collected on the Wombat High‐Intensity Powder Diffractometer of the Australian Nuclear Science and Technology Organisation (ANSTO). Detailed information on both crystal and magnetic structure was obtained from Rietveld refinements of NPD data using the FullProf program. In addition, cryogenic ^57^Fe Mössbauer spectroscopy experiments were also carried out on a low‐temperature closed cycle Fe‐57 Mössbauer spectrometer to help determine the hyperfine magnetic structure.

## Conflict of Interest

The authors declare no conflict of interest.

## Supporting information

Supporting Information

## Data Availability

The data that support the findings of this study are available from the corresponding author upon reasonable request.
