# Supplementary material for: Significantly Promoting the Thermal Conductivity and Machinability of Negative Thermal Expansion Alloy via In Situ Precipitation of Copper Networks
Source: Adv Sci (Weinh). 2024 Aug 28;11(40):2404838. doi: 10.1002/advs.202404838 (PMC11515899; doi:10.1002/advs.202404838)
Supplement: Supplementary file 1 — Supporting Information [file ADVS-11-2404838-s001.docx]

Supplementary materials for

**Significantly Promoting the Thermal Conductivity and** **Machinability of Negative Thermal Expansion Alloy via In Situ Precipitation of Copper Networks**

*Minjun Ai, Yuzhu Song^*^, Feixiang Long, Yuanpeng Zhang, Ke An, Dunji Yu, Yan Chen, Yuki Sakai, Masahito Ikeda, Kazuki Takahashi, Masaki Azuma, Naike Shi, Chang Zhou^*^, Jun Chen*

M. J. Ai, Dr. Y. Z. Song, F. X. Long, Prof. C. Zhou, Prof. J. Chen

Department of Physical Chemistry, Beijing Advanced Innovation Center for Materials Genome Engineering, University of Science and Technology Beijing, Beijing 100083, China

E-mail: yuzhusong@ustb.edu.cn; changzhou@ustb.edu.cn

Dr. Y. P. Zhang, Dr. K. An, Dr. D. J. Yu, Dr. Y. Chen

Neutron Scattering Division, Oak Ridge National Laboratory, Oak Ridge, Tennessee 37831, United States

Dr. Y. Sakai, Prof. M. Azuma

Kanagawa Institute of Industrial Science and Technology (KISTEC), 705-1 Shimoimaizumi, Ebina, Kanagawa 243-0435, Japan

Dr. Y. Sakai, Dr. M. Ikeda, Dr. K. Takahashi, Prof. M. Azuma

Laboratory for Materials and Structures, Institute of Innovative Research, Tokyo Institute of Technology, Yokohama, 226–8503, Japan

Dr. C. Zhou

State Key Laboratory for Advanced Metals and Materials, University of Science and Technology Beijing, Beijing 100083, China

Prof. J. Chen

Hainan University, Haikou 570228, Hainan Province, China

Keywords: negative thermal expansion, high thermal conductivity, eutectic precipitation, copper networks


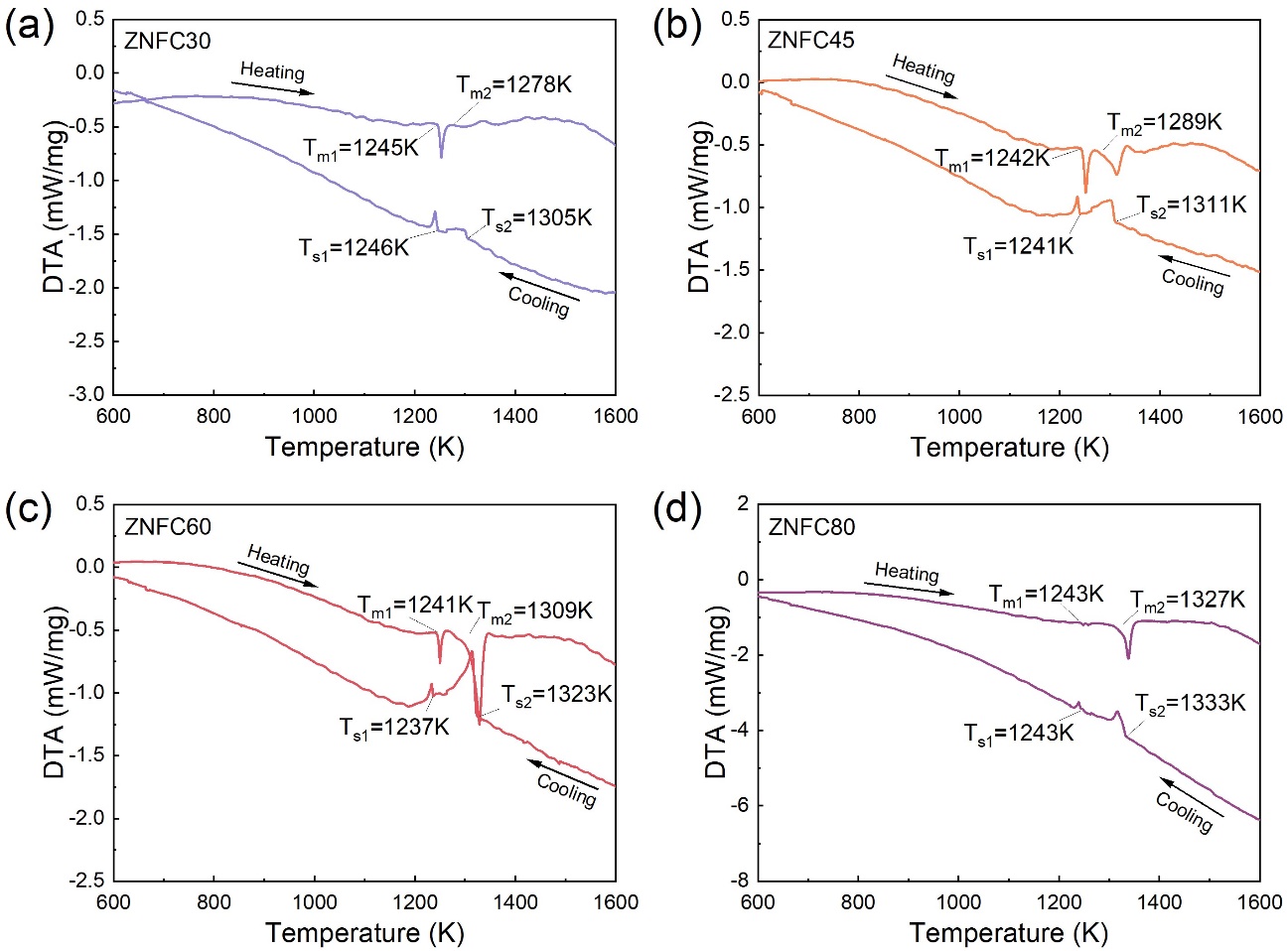


Figure S1. TG-DTA curves of (a) ZNFC30, (b) ZNFC45, (c) ZNFC60, and (d) ZNFC80.

Two consecutive melting and corresponding solidification events can be observed in the TG-DTA curves. The first melting (solidification) event exhibits a sharp endothermic (exothermic) peak with melting point (T_m1_, as well as solidification point, T_s1_) close to 1243 K for each sample, characterized as a eutectic reaction. The endothermic (exothermic) peak of the second melting (solidification) event extends broader on the side connected to the eutectic reaction. Also, the melting point (T_m2_) and corresponding solidification point (T_s2_) of this process increase with the rise in copper content, indicating a hypereutectic process. Additionally, the weak hypereutectic peak that nearly disappears in the TG-DTA curve of ZNFC30 manifests that its composition is close to the eutectic point.


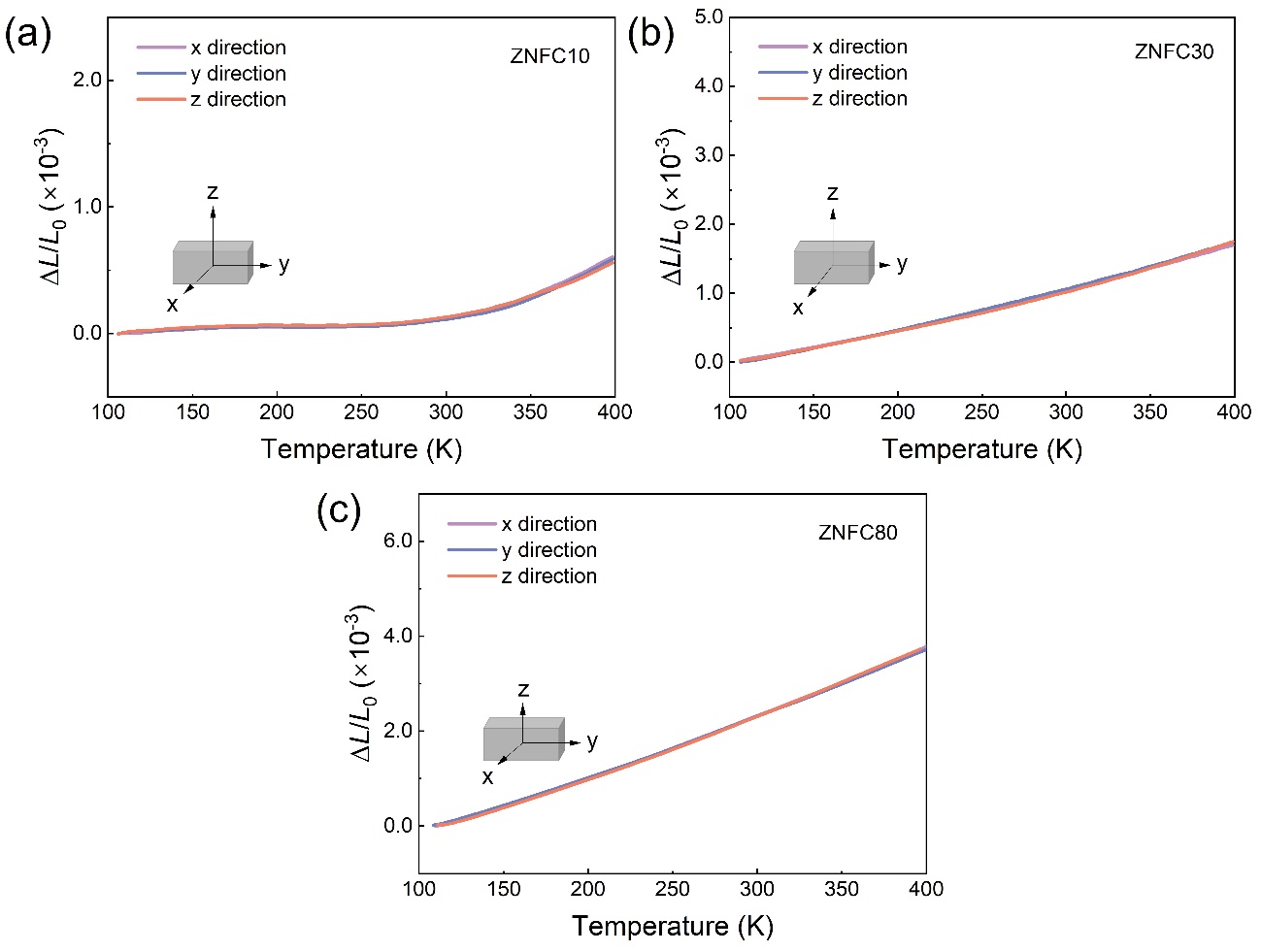


Figure S2. Linear thermal expansion curves of (a) ZNFC10, (b) ZNFC30, and (c) ZNFC80 samples along three orthogonal directions.

**Details of thermal conductivity measurements**

The one-dimensional steady-state heat flow method was adopted to measure the thermal conductivity of the samples. The measurements were based on the Fourier's law:

*Q = - λ * A ** $\frac{\text{dT}}{\text{dx}}$ (1)

where *Q* is the heat flow, *λ* is the thermal conductivity, *A* is the cross-sectional area, and $\frac{\text{dT}}{\text{dx}}$ is the temperature gradient in the direction of heat conduction.

During testing, one end of the sample was heated while the other was kept cold to generate a temperature gradient. The samples were fabricated into long prisms (5 mm * 5 mm * 20 mm) to ensure the one-dimensional heat flow and $\frac{\text{dT}}{\text{dx}}$ can be replaced by $\frac{\text{ΔT}}{\text{Δx}}$. The *Q* and *∆T* can be tested and calibrated by thermal conductivity meter, and *λ* can be calculated accordingly. With steady states well achieved, the thermal conductivity meter CTM-60 has a measurement error of less than 5%. To validate the accuracy of the test results, the thermal conductivity of pure copper was tested and found to be 399.1 W m^-1^ K^-1^ at 297 K.


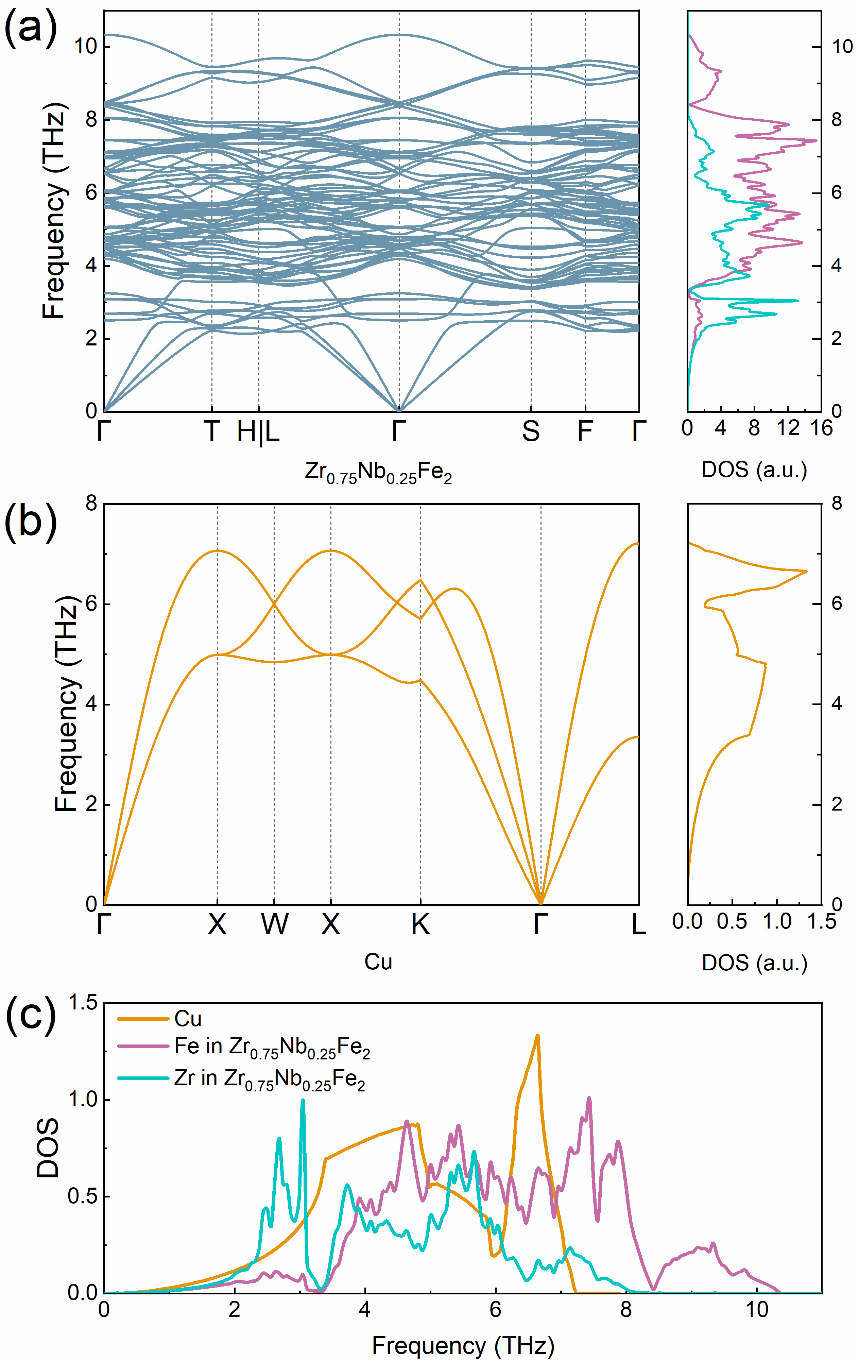


Figure S3. (a) Phonon dispersion curves and corresponding DOS of Zr_0.75_Nb_0.25_Fe_2_. (b) Phonon dispersion curves and corresponding DOS of Cu. (c) Normalized DOS Comparison of Zr_0.75_Nb_0.25_Fe_2_ and Cu.

The phonon vibration frequencies of Zr_0.75_Nb_0.25_Fe_2_ and copper are both within 10 THz, and the DOS peaks of Cu atoms also overlap with that of Fe and Zr/Nb in Zr_0.75_Nb_0.25_Fe_2_. The previous researches have proved that the microscale heat transfer interfaces wouldn’t lead to significant shifts in phonon frequencies^[1-3]^. Therefore, it can be concluded that the phonon mismatch between eutectic copper lamellae and (Zr,Nb)Fe_2_ is low. However, it’s noteworthy that the thermal conductivity of Zr_0.7_Nb_0.3_Fe_2_ is 7.82 W m^-1^ K^-1^, less than 2% of that of copper. The entire heat transfer process will be hindered on the (Zr, Nb)Fe_2_ side, regardless of whether the thermal conduction between the two phases behaves well. Therefore, when eutectic copper lamellae interconnect as copper networks, a relatively higher heat transfer channel will contribute to facilitate the thermal conductivity of the dual-phase alloy^[4, 5]^.

Table S1. The thermal conductivity, CTE, and mechanical properties of Zr-Nb-Fe-Cu alloys compared with the representative LTE/ZTE/NTE alloys.

| LTE/ZTE/NTE  alloys | Thermal conductivity  (W m^-1^ K^-1^) | Linear CTE  (×10^-6^ K^-1^) | Mechanical properties | | Data sources |
| --- | --- | --- | --- | --- | --- |
|  |  |  | Compressive stress  (MPa) | Compressive strain  (%) |  |
| ZNFC30 | 23.64 | 5.72 | 554 | 1.77 | This work |
| LaFe_10.6_Si_2.4_ | 6 | -0.8 | - | - | [6, 7] |
| LaFe_11_Si_2_ hydride | 0.5 | 6 | - | - | [8] |
| Mn_3_(Cu_0.2_Ni_0.4_Ge_0.4_)N | 1.6 | 0.6 | - | - | [9] |
| Gd_0.25_Dy_0.75_Co_1.93_Fe_0.07_ | 6.26 | 0.61 | - | - | [10] |
| Hf_0.87_Ta_0.13_Fe_2_ | 10 | -16.3 | 382 | 2.7 | [11] |
| Hf_0.87_Ta_0.13_Fe_3_ | 15.6 | 3.3 | - | - | [12] |
| NiTi | 18 | 10 | ~400 (Tensile) | - | [13] |
| Tb(Co_1.9_Fe_0.1_) | 6.3 | 0.48 | - | - | [14] |
| Mn_0.98_CoGe | 6.4 | -141 | 60 | 3.6 | [15, 16] |
| Hf-Ta-Fe-Cu | 21.9 | 1.7 | 1040 | 9 | [17] |
| Zr_0.7_Nb_0.3_Fe_2_ | 7.82 | -2.02 | - | - | This work |
| Invar | 13.84 | 0.62 | 490 (Tensile) | 40 (Tensile) | [18, 19] |
| Kovar | 16 | 5.87 | - | - | [18] |

Table S2. Results of Rietveld structure refinements of Zr-Nb-Fe-Cu alloys.

| Samples | Phase fractions (wt. %) | | Lattice parameters (Å) | |
| --- | --- | --- | --- | --- |
|  | (Zr,Nb)Fe_2_ | Copper | (Zr,Nb)Fe_2_ | Copper |
| ZNFC10 | 96.85 | 3.15 | 6.991 | 3.618 |
| ZNFC20 | 85.45 | 14.55 | 6.993 | 3.617 |
| ZNFC30 | 63.36 | 36.64 | 7.012 | 3.616 |
| ZNFC45 | 47.99 | 52.01 | 7.029 | 3.616 |
| ZNFC60 | 29.01 | 70.99 | 7.022 | 3.616 |
| ZNFC80 | 13.37 | 86.63 | 7.062 | 3.616 |

It’s worth noting that the lattice parameters of (Zr,Nb)Fe_2_ increases slightly with the phase fractions of the copper. The previous research has proved that the increase of Zr/Nb ratio would lead to a significant enlargement of the (Zr,Nb)Fe_2_ unit cell^[20]^. We have concluded that (Section **2.4** in manuscript) the eutectic copper phase absorbs parts of Zr and Nb atoms from (Zr,Nb)Fe_2_, resulting in a deviation of the ratio of Zr/Nb from 7:3. Therefore, we deem that the increase of lattice parameters results from the increase of Zr/Nb ratio.


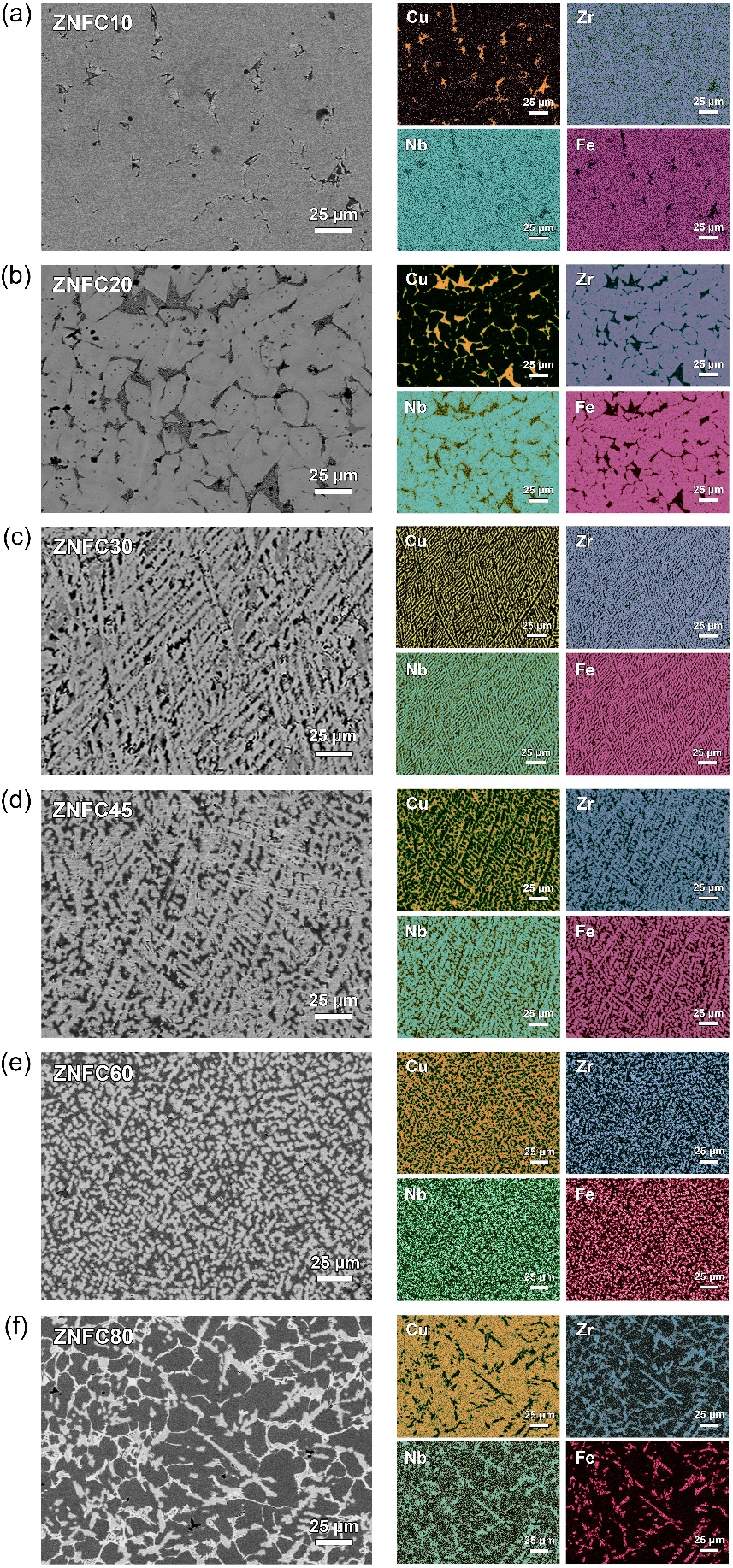


Figure S4. BSE images and corresponding EDS mappings of (a) ZNFC10, (b) ZNFC20, (c) ZNFC30, (d) ZNFC45, (e) ZNFC60, and (f) ZNFC80.


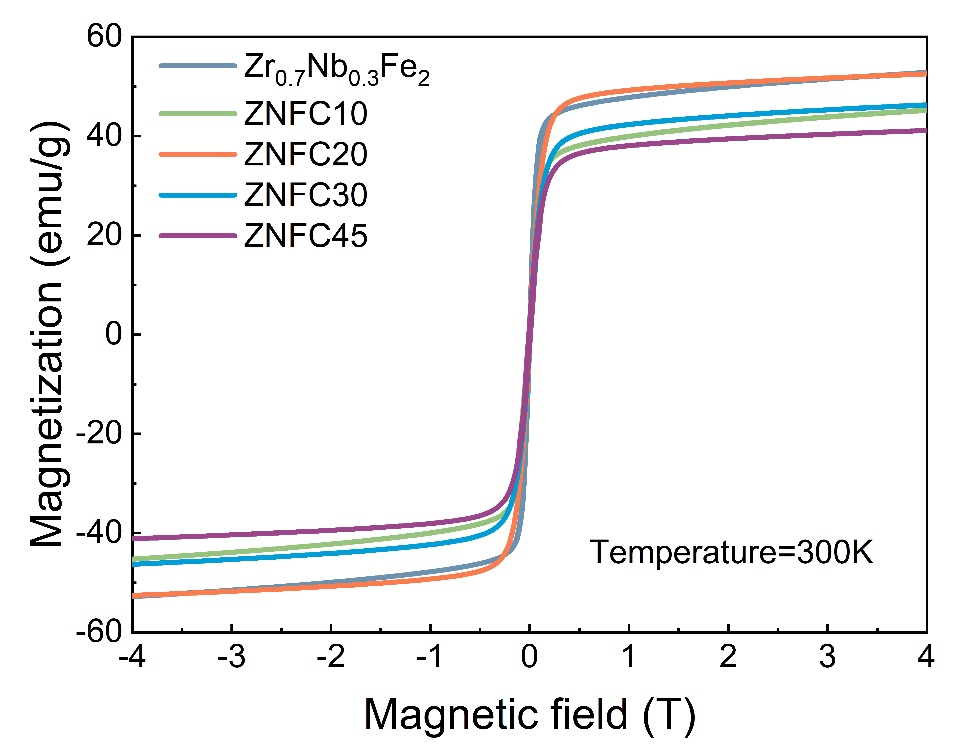


Figure S5. Magnetization curves as a function of magnetic field for Zr-Nb-Fe-Cu alloys at 300 K.


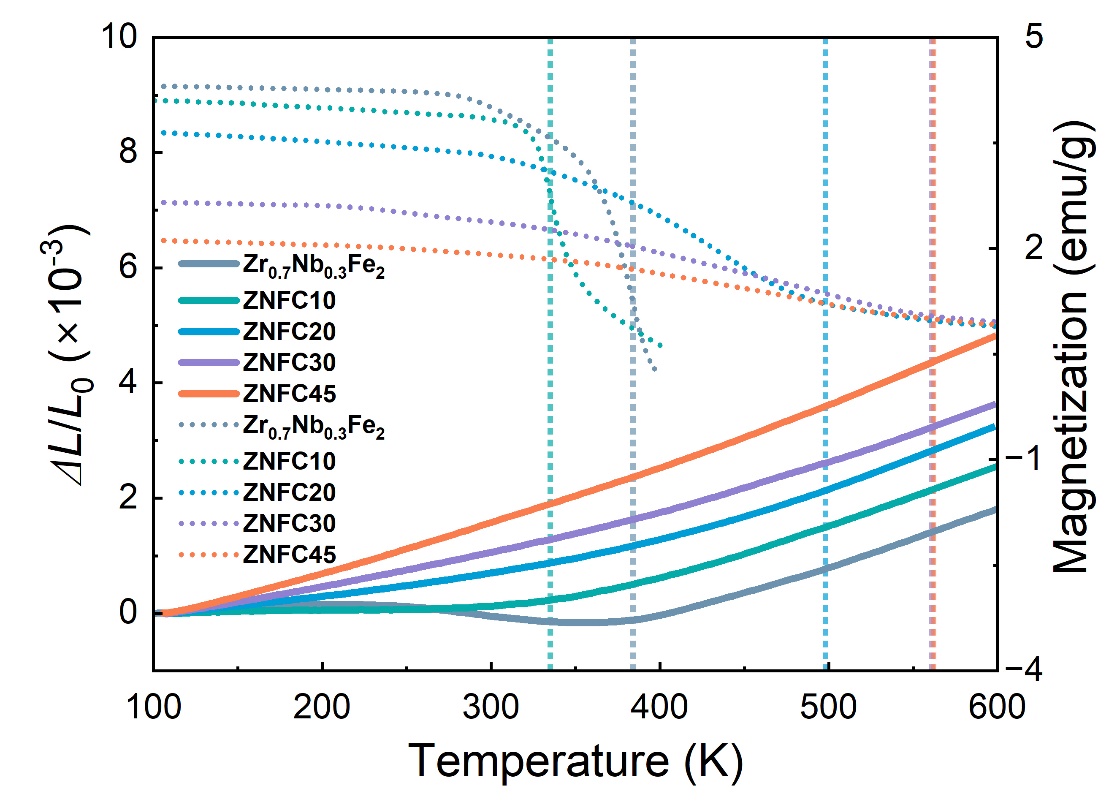


Figure S6. A comparison of temperature-dependent thermal expansion curves (solid lines) and magnetization curves (dotted lines) of Zr-Nb-Fe-Cu alloys.


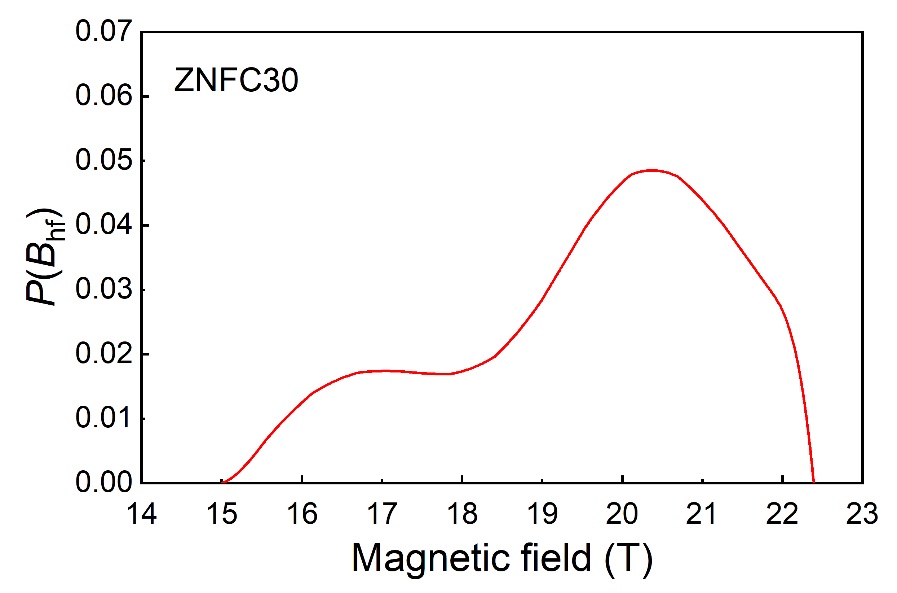


Figure S7. Distribution of hyperfine magnetic field of Mössbauer spectrum acquired for ZNFC30 at 6.2 K.

Table S3. Fitting results of Mössbauer spectrum acquired for ZNFC30 at 6.2 K.

| Sites | Fe at 16d of Laves | | Fe in solid solution |
| --- | --- | --- | --- |
| Isomer shift (mm/s) | -0.040 | 0 | |
| Quadrupole splitting (mm/s) | 0.037 | 0 | |
| Magnetic field (T) | 19.58 | 11.08 | |
| Line width (mm/s) | 0.52 | 1.01 | |





Figure S8. DOS of atoms in Zr_0.75_Nb_0.25_Fe_2_ compared with Zr_0.75_Nb_0.25_(Fe,Cu)_2_.


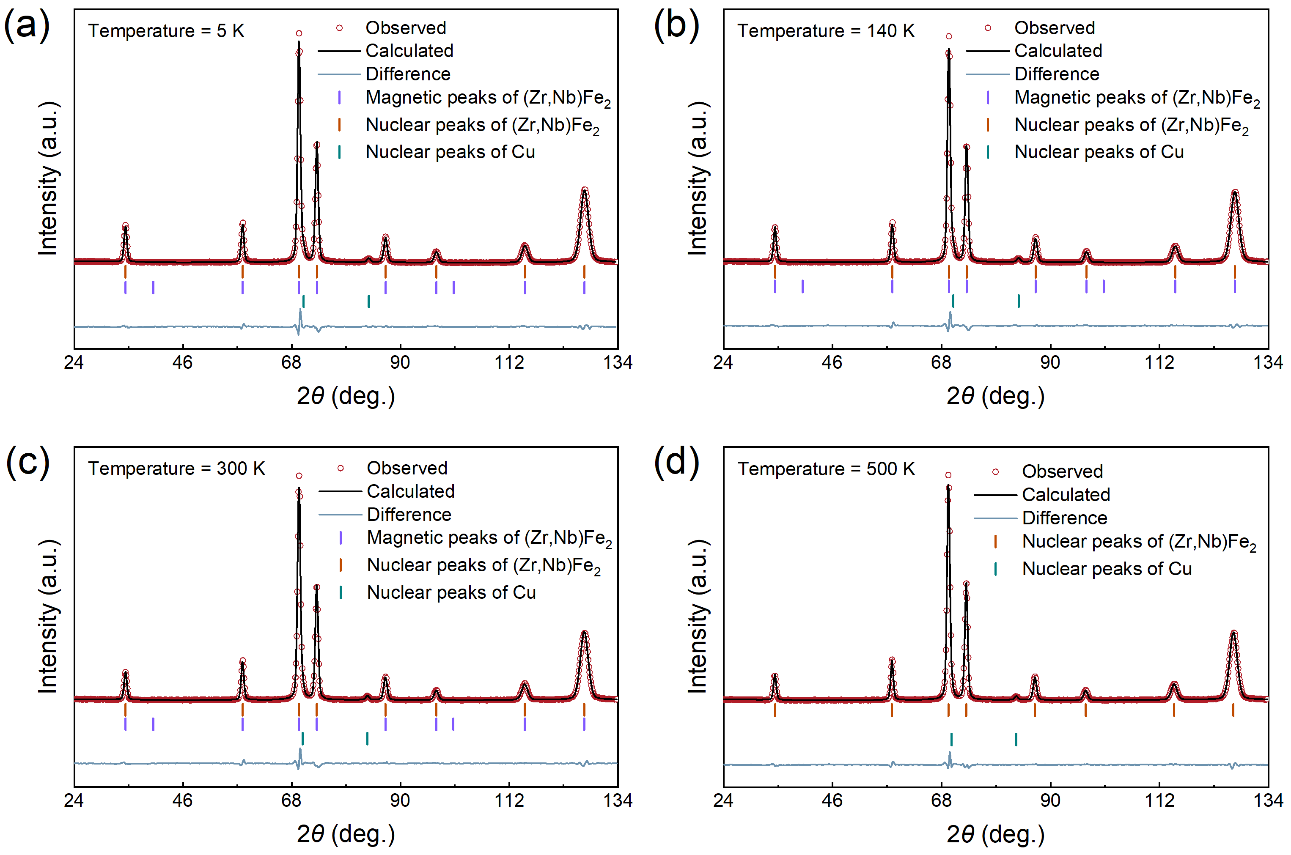


Figure S9. Magnetic structure refinements of NPD patterns of ZNFC10 at (a) 5 K, (b) 140 K, (c) 300 K, and (d) 500 K (higher than *T*_c_).

Table S4. Site occupation results of Rietveld magnetic structure refinements of ZNFC10.

| Temperature (K) | Site occupation of (Zr,Nb)Fe_2_ | | | |
| --- | --- | --- | --- | --- |
|  | 8a | | 16d | |
| 500 | Zr | 0.7 | Fe | 1.86 |
|  | Nb | 0.3 | Cu | 0.14 |


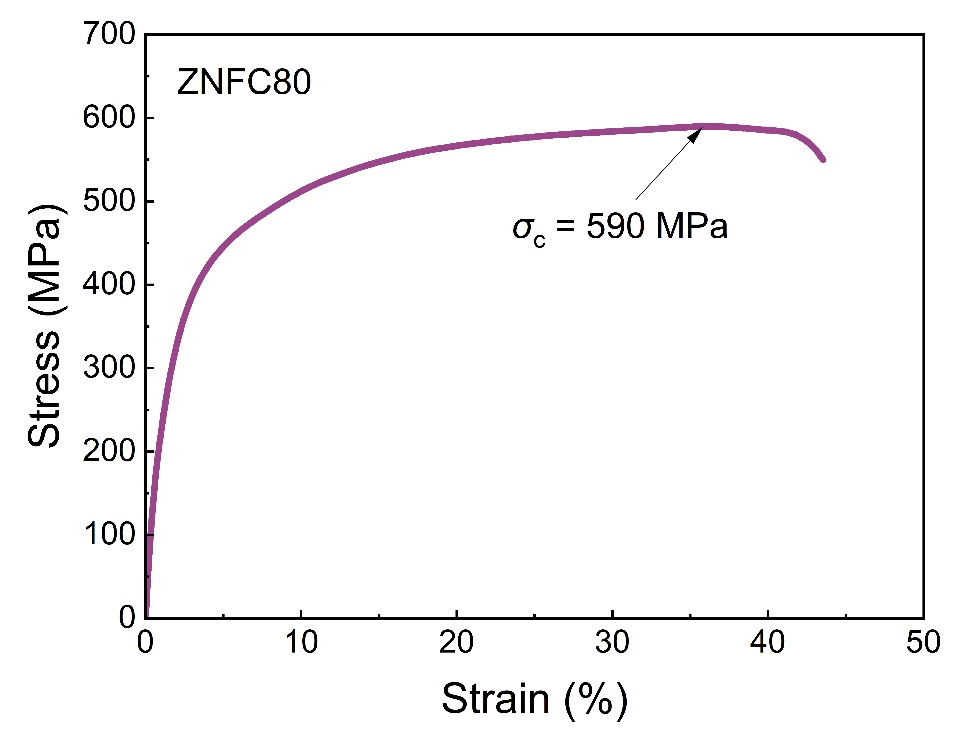


Figure S10. Compressive stress-strain curves of ZNFC80 at room temperature.





Figure S11. (a) Thermal expansion curves, (b) thermal conductivity, and (c) compressive stress-strain curves of ZNFC30 after 50 and 100 high-low temperature cycles compared with those of initial sample.

The ability to maintain performance after thermal cycling is a crucial indicator when considering the practical application of materials in electronic packaging. Based on the highly fine and uniform lamellae structure and the semi-coherent interfaces of the alloy, we believe it will remain stable and maintain the performances after high-temperature thermal cycling. We have conducted high-low temperature cycling experiments for 50 and 100 cycles on the optimal ZNFC30 (Figure S11). The low temperature environment was provided by liquid nitrogen (77 K) and the high temperature environment was provided by heated dimethyl silicone oil (530 K). The test results show differences merely within the range of testing errors compared with those before cycling, indicating no performance degradation after 100 cycles. Therefore, the thermal conductivity, thermal expansion, and mechanical properties of the alloys can remain stable after high-low temperature thermal cycling.

**Application of Zr-Nb-Fe-Cu alloys in electronic packaging**

The rapid development of highly integrated and miniaturized integrated circuits (IC) has significantly increased their heat generation. The lifespan of electronic components decreases significantly with the rapid temperature rise. The electronic packaging materials, which directly contact the chips (such as Si, Ge, GaAs with low CTE approximately 5 ppm/K), will lead to thermal stresses and cause electronic device failures if the CTEs are mismatched. Therefore, designing electronic packaging materials with low thermal expansion and high thermal conductivity has become one of the primary challenges faced by the IC industry.

The Zr-Nb-Fe-Cu alloys will be primarily used for second-level packaging materials (as shown in Figure 1a). During the soldering process in packaging and subsequent operation of electronic devices, significant heat fluxes will be generated across the chips. The package substrates which are directly connected to the chips should promptly dissipate the heat to prevent excessively high temperature. Additionally, the chips also expand when heated up, and the package substrates should expand synchronously to mitigate the thermal stress arising from CTE mismatch. Therefore, both high thermal conductivity and chip-matched CTE (low CTE) are highly desired for electronic packaging materials.

**References**

[1] A. Giri, P. E. Hopkins, *Adv. Funct. Mater.* **2019**, *30*, 1903857.

[2] L. Chen, S. Chen, Y. Hou, *Carbon* **2019**, *148*, 249.

[3] C. Zhang, W. Chen, Y. Tao, W. Zhao, S. Cai, C. Liu, Z. Ni, D. Xu, Z. Wei, J. Yang, K. Bi, Y. Chen, *Carbon* **2017**, *115*, 665.

[4] X. Miao, C. Wang, T. Liao, S. Ju, J. Zha, W. Wang, J. Liu, Y. Zhang, Q. Ren, F. Xu, L. Caron, *Acta Mater.* **2023**, *242*, 118453.

[5] F. Xu, Y. Cui, D. Bao, D. Lin, S. Yuan, X. Wang, H. Wang, Y. Sun, *Chem. Eng. J.* **2020**, *388*, 124287.

[6] W. Wang, R. Huang, W. Li, J. Tan, Y. Zhao, S. Li, C. Huang, L. Li, *PCCP* **2015**, *17*, 2352.

[7] R. J. Huang, Y. Y. Liu, W. Fan, J. Tan, F. R. Xiao, L. H. Qian, L. F. Li, *J. Am. Chem. Soc.* **2013**, *135*, 11469.

[8] S. Li, R. Huang, Y. Zhao, W. Wang, Y. Han, L. Li, *Adv. Funct. Mater.* **2016**, *27*, 1604195.

[9] R. Huang, X. Chu, Z. Wu, L. Li, X. Xu, *Appl. Phys. A* **2010**, *99*, 465.

[10] J. Hu, K. Lin, Y. Cao, C. Yu, W. Li, R. Huang, H. E. Fischer, K. Kato, Y. Song, J. Chen, H. Zhang, X. Xing, *Inorg. Chem.* **2019**, *58*, 5401.

[11] L. F. Li, P. Tong, Y. M. Zou, W. Tong, W. B. Jiang, Y. Jiang, X. K. Zhang, J. C. Lin, M. Wang, C. Yang, X. B. Zhu, W. H. Song, Y. P. Sun, *Acta Mater.* **2018**, *161*, 258.

[12] L. Li, P. Tong, W. Tong, W. Jiang, Y. Ding, H. Lin, J. Lin, C. Yang, F. Zhu, X. Zhang, X. Zhu, W. Song, Y. Sun, *Inorg. Chem.* **2019**, *58*, 16818.

[13] J. A. Shaw, *Int. J. Plast.* **2000**, *16*, 541.

[14] Y. Z. Song, J. Chen, X. Z. Liu, C. W. Wang, J. Zhang, H. Liu, H. Zhu, L. Hu, K. Lin, S. T. Zhang, X. R. Xing, *J. Am. Chem. Soc.* **2018**, *140*, 602.

[15] F. Zhu, J. C. Lin, W. B. Jiang, C. Yang, L. F. Li, X. K. Zhang, W. H. Song, X. B. Zhu, P. Tong, Y. P. Sun, *Scr. Mater.* **2018**, *150*, 96.

[16] J. Lin, P. Tong, K. Zhang, H. Tong, X. Guo, C. Yang, Y. Wu, M. Wang, S. Lin, L. Chen, W. Song, Y. Sun, *Appl. Phys. Lett.* **2016**, *109*, 241903.

[17] L. F. Li, P. Tong, W. B. Jiang, J. C. Lin, F. Zhu, M. F. Shu, Z. T. Fang, G. C. Zhao, Z. Z. Jiang, W. Wang, C. B. Pan, X. B. Zhu, W. H. Song, Y. P. Sun, *Materialia* **2020**, *9*, 100637.

[18] M. G. Pecht, R. Agarwal, P. McCluskey, T. Dishongh, S. Javadpour, R. Mahajan, *Electronic Packaging: Materials and Their Properties*, CRC press, Boca Raton, FL, USA 1998.

[19] J. Hidalgo, A. Jiménez-Morales, T. Barriere, J. C. Gelin, J. M. Torralba, *Powder Metall.* **2013**, *57*, 127.

[20] Y. Z. Song, Q. Sun, T. Yokoyama, H. H. Zhu, Q. Li, R. J. Huang, Y. Ren, Q. Z. Huang, X. R. Xing, J. Chen, *J. Phys. Chem. Lett.* **2020**, *11*, 1954.
